# Chronic expanding hematoma in the liver: a case report

**DOI:** 10.1186/s40792-022-01548-w

**Published:** 2022-11-22

**Authors:** Atsuki Taniguchi, Takeyoshi Nishiyama, Jun Kozai, Izuru Endo, Nobuyuki Watanabe

**Affiliations:** Department of Surgery, Mitoyo General Hospital, 809, Toyohama-Cho Himehama, Kanonji, Kagawa 769-1695 Japan

**Keywords:** Chronic expanding hematoma, Liver, Rib fracture, Hepatectomy

## Abstract

**Background:**

A hematoma that gradually increases over a chronic course of months or longer is defined as a chronic expanding hematoma (CEH). CEHs often develop in the limbs and on body surfaces that are susceptible to external stimuli. CEHs in the intrathoracic or intraperitoneal organs are uncommon, with liver CEHs being particularly rare worldwide.

**Case presentation:**

A 57-year-old woman was previously diagnosed with a giant cyst in the right liver lobe, with a longer axis of approximately 15 cm. Abdominal ultrasonography findings suggested a complex cyst, and she was referred to our hospital for further inspection. Although CEH was suspected, it was difficult to exclude malignant diseases such as intraductal papillary neoplasm of the bile duct and cystadenocarcinoma. There was a possibility of malignant disease and the exclusion of surrounding organs due to tumor growth. Therefore, a right hepatectomy was performed. Pathological examination revealed a pseudocyst containing a clot, which was consistent with CEH.

**Conclusions:**

CEH rarely occurs in the liver; however, it is necessary to consider CEH when a slow-growing hepatic mass that shows a mosaic pattern on magnetic resonance imaging is found.

## Background

Hematoma may form in various organs due to trauma, surgery, and oral anticoagulant or antiplatelet drugs, but most are eventually naturally absorbed. In contrast, a hematoma that gradually grows over a chronic course for more than 1 month is defined as a chronic expanding hematoma (CEH). As a CEH grows, it leads to exclusion symptoms depending on its location. They often develop on the limbs and body surface that are exposed to external stimuli, but rarely in intrathoracic or intraperitoneal organs. We report a case of a CEH that occurred in the liver.

## Case presentation

**Fig. 1:**
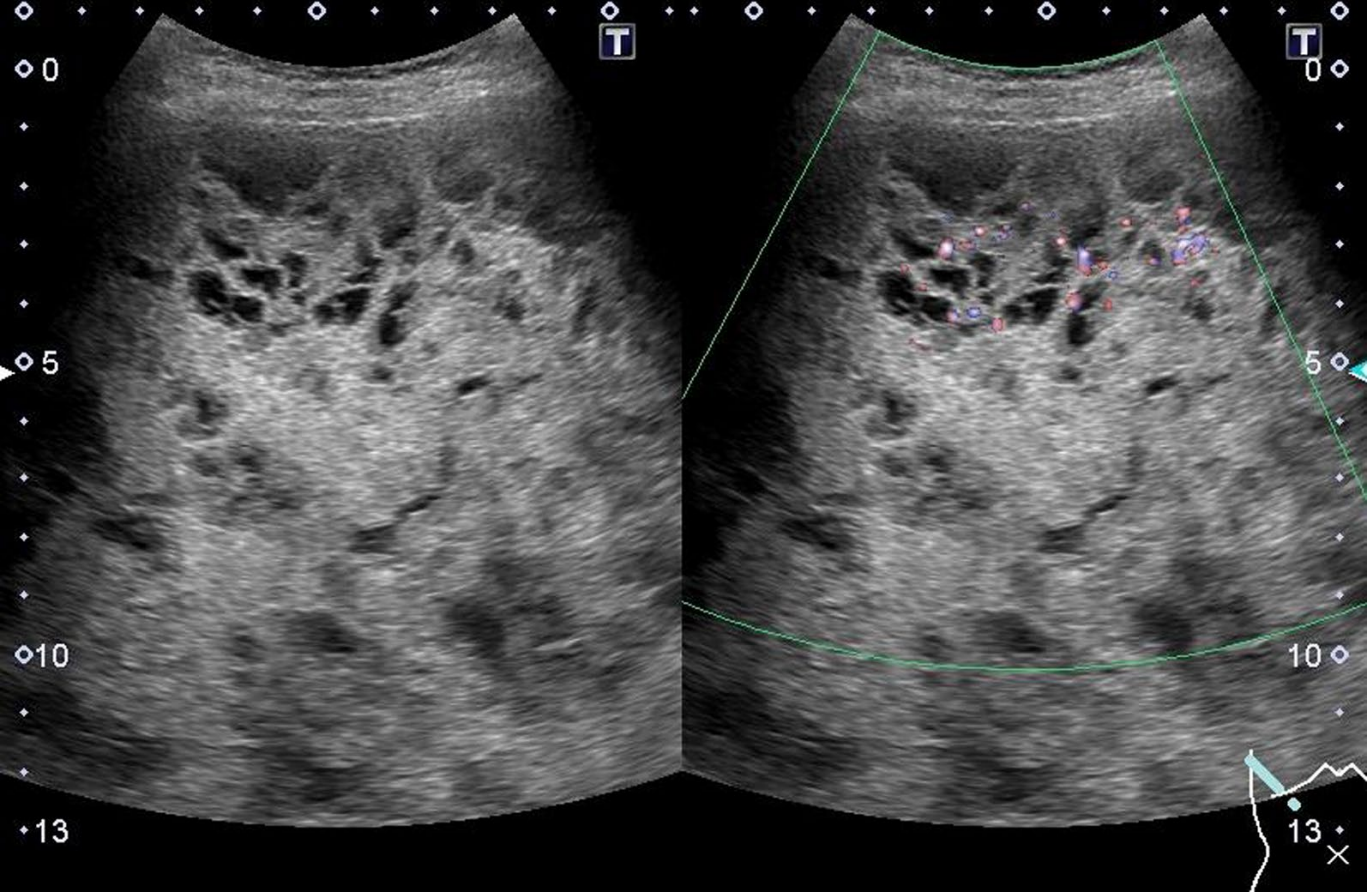
Abdominal ultrasonography. The inside of the tumor was recognized as a structure divided into many spaces, and blood flow was confirmed in the septum

**Fig. 2:**
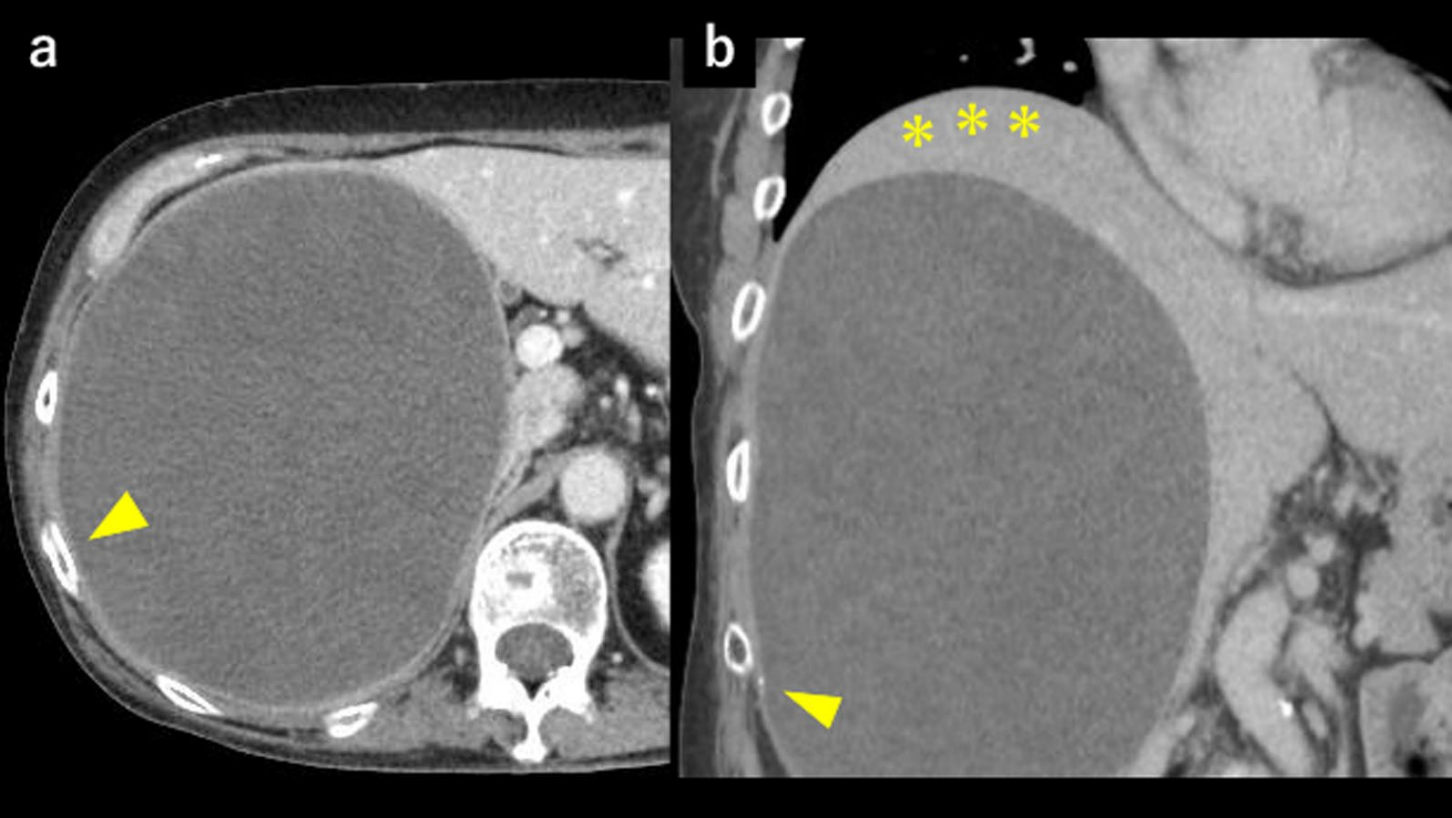
Contrast-enhanced CT (portal vein phase). **a** Huge mass with low absorption occupied most of the right lobe of the liver, which had focal enhancement on the tumor margin. An old right 9th rib fracture was observed (arrow). **b** Fine calcification was observed at the tumor margin (arrow). The right diaphragm was elevated due to the tumor (*)

**Fig. 3:**
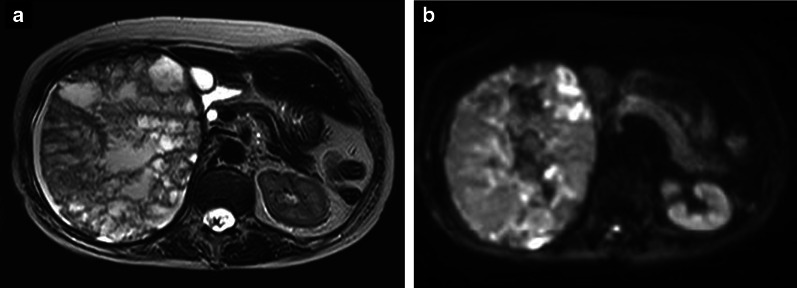
MRI. **a** T2-weighted imaging. The inside of the tumor showed a mosaic pattern with a mixture of high and low signals. **b** Diffusion weighted imaging: *b* = 1000. A high signal was observed predominantly at the tumor margin

**Fig. 4:**
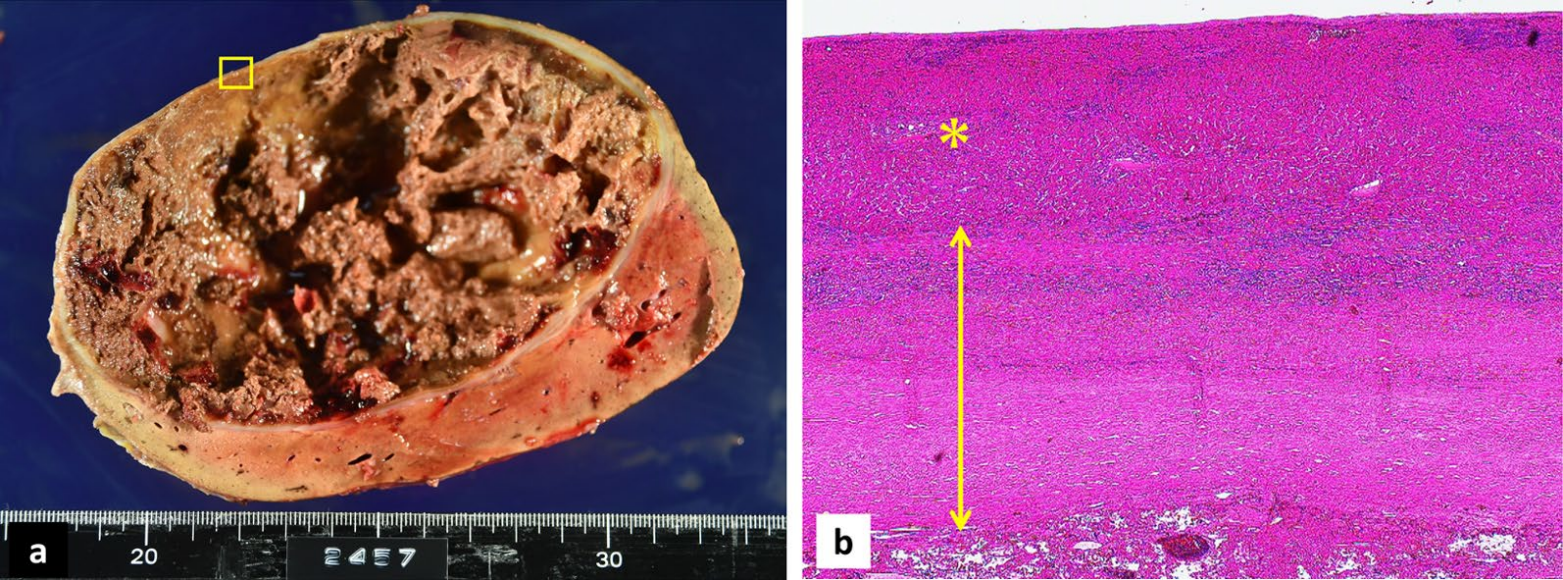
Pathological findings. **a** Tumor was covered with a fibrous cap, and a clot was found inside. **b** Tumor edge (□) in **a** is enlarged, the cyst wall consisted of vitrified connective tissue, and no covering epithelium was confirmed (double arrow). It is covered with thinly stretched liver parenchyma (*)

The patient was a 57-year-old woman who previously presented with a giant liver mass on abdominal ultrasonography during a medical examination. Detailed computed tomography (CT) showed non-uniformity inside the tumor and gradual growth. The patient visited our hospital for further detailed examination and treatment. Blood examination findings were as follows: hemoglobin 14.5 g/dl, platelets 28.5 × 10^4^/µl, prothrombin time/international normalized ratio 1.02, activated partial thromboplastin time 27.7 s, aspartate aminotransferase 41 U/L, alanine aminotransferase 18 U/L, lactate dehydrogenase 265 U/L, total bilirubin 0.7 mg/dl. In addition, tumor markers such as carcinoembryonic antigen, carbohydrate antigen 19-9, protein induced by vitamin K absence or antagonist-II, and α-fetoprotein were within their respective normal ranges. Liver damage was grade A, and Child–Pugh grade was A. Abdominal ultrasonography revealed that the inside of the tumor was divided into many spaces, and blood flow was confirmed in the septum (Fig. 1). Contrast-enhanced CT (Fig. 2a, b) showed that a large mass with a non-uniform interior occupied most of the right lobe of the liver. Focal enhancement and fine calcification on the margin of the mass was observed. An old fracture of the right 9th rib due to a previous traumatic road traffic accident was observed. Elevation of the right diaphragm and exclusion of the inferior vena cava (IVC) due to the large mass were also noted. Magnetic resonance imaging (MRI) (Fig. 3a, b) showed a mosaic pattern with a mixture of high and low signals in the tumor on T2 weighted images, and a high signal at the tumor margin on diffusion-weighted images. The respiratory function test showed a restraining pattern. Based on the above laboratory findings, CEH was suspected to be the most likely diagnosis, but other malignant diseases could not be ruled out. Since the patient exhibited exclusion symptoms due to tumor growth, a right hepatectomy was scheduled. Intraoperatively, a tight cystic lesion in the right lobe of the liver and a wide adhesion to the hepatic flexure at the inferior margin of the tumor were observed. Standard right hepatectomy was performed. The operative time was 257 min, and the intraoperative blood loss was 650 ml. The weight of the resected liver was 2760 g. On macroscopic examination, the tumor was covered with a fibrous cap, and a clot was found inside (Fig. 4a). Histopathological findings showed a pseudocapsule-containing blood component. The cyst wall consisted of a vitrified connective weave. The cyst was covered with thinly stretched liver parenchyma only, with no covering epithelium (Fig. 4b).

## Discussion

Reid et al. were the first to report a CEH, a hematoma that occurs after trauma or surgery and develops gradually over 1 month or more [1]. Pathologically, no neoplastic changes are observed, and such hematomas may occur due to the use of anticoagulants or antiplatelet drugs, even in the absence of previous trauma. The mechanisms underlying the onset and development of CEHs remain unclear; however, hypothetically, they occur when a hematoma caused by trauma is not absorbed, and damaged cells, such as blood cells, cause chronic inflammation that leads to the formation of a prominent capsule around the hematoma. The leaking blood forms vessels inside the hematoma and gradually increases its size [2]. In our case, there was no history of oral anticoagulants or antiplatelet drugs, indicating that CEH might have occurred after trauma. The patient had a history of rib fracture due to a traumatic traffic accident. Considering the clinical presentation of the CEH and the positional relationship between its location and the previous rib fracture, its development may have been associated with the traumatic traffic accident. We reviewed the patient’s history of visits to clinics other than our own and assessed the course of the hematoma’s volume. The change in volume from 1.59 × 10^3^ cm^3^ (at 1651 days prior to the operation) to 2.20 × 103 cm^3^ (at 60 days prior to the operation) is indicative of slow growth. However, a causal relationship between the traumatic accident and the CEH occurring in the liver could not be clearly established, because the initial CT images at the time of the traffic accident could not be obtained.

CEH often occurs in palpable areas, such as the body surface and limbs, but in theory, it can occur subsequent to bleeding in any organ. However, the published reports show a clear bias in the organs in which it develops, where it is relatively rare to occur in the intrathoracic or intraperitoneal organs. We report a rare occurrence of CEH in the liver. From 1968 to 2021, we queried PubMed for the keyword “chronic expanding hematoma”, finding 90 confirmed cases. Eleven cases occurred in the retroperitoneum and intraperitoneal organs (Table 1). Among them, CEH in the liver was reported in only one case [3], with our case being the second reported worldwide. In our case, no antiplatelet or anticoagulant medications were used and there was no history of trauma.

**Table 1:** Reported cases of CEH occurring in the retroperitoneum and intraperitoneal organs

Reports (year)	Age/Sex	Location	Treatment	Cause	Size
Syuto (2013)	69/M	Retroperitoneum	Incomplete resection	NA	200 mm
Kubota (2015)	72/M	Retroperitoneum	Incomplete resection	Antiplatelet drug	200 mm
Yamada (2003)	59/M	Lt. adrenal gland	Complete resection (Lt. adrenalectomy + partial nephrectomy)	Antiplatelet drug	120 mm
Asayama (1998)	35/F	Spleen	Complete resection (splenectomy)	Bleeding of splenic AML	NA
Kajioka (2020)	68/M	Lt. adrenal gland	Complete resection (Lt. adrenalectomy + DP + partial transverse mesocolon resection)	Antiplatelet drug	250 mm
Sunada (2015)	67/M	Lt. adrenal gland	Complete resection (Lt. adrenalectomy)	Anticoagulant, antiplatelet drug	166 mm
Kaneko (2009)	34/F	Retroperitoneum	Complete resection (Rt. adrenalectomy + partial hepatectomy)	NA	120 mm
Ono (2021)	56/M	Liver	CT guided drainage, TAE of feeder artery → Complete resection (Rt. hepatectomy)	NA	175 mm
Yamasaki (2005)	53/M	Iliopsoas	Complete resection (tumor excision)	Trauma	120 mm
Sakurai (2010)	72/F	Peritoneal cavity	Complete resection (tumor excision)	Post-caesarean section	100 mm
Masuda (2020)	72/F	Peritoneal cavity	Complete resection (tumor excision)	Traffic trauma	270 mm
Our case (2021)	57/F	Liver	Complete resection (Rt. hepatectomy)	Traffic trauma (rib fracture)	150 mm

*DP* distal pancreatectomy, *TAE* transcatheter arterial embolization, *NA* not available, *AML* angiomyolipoma

Given the paucity of available reports, it is difficult to exclude malignant diseases when CEH occurs in the organs. MRI is useful for the differential diagnosis of malignant diseases [4]. Since CEH is characterized by a mixture of old and fresh bleeding inside the cyst, MRI findings show a mosaic pattern. Positron emission tomography–CT is not always useful for ruling out malignant diseases, since some studies have reported fluorodeoxyglucose accumulation in the tumor margin, reflecting inflammation [5, 6]. The degree of inflammation of the cyst wall varies from case to case. Some cases partially form a xanthogranuloma [6, 7]. Fine calcification is often found at the hematoma margin, suggesting that calcification is indicative of previous inflammation. However, calcification is not always seen in such cases and may be due to other causes, since it is not observed in cases with a xanthogranuloma within the CEH. Calcification alone may not be a reflection of the intensity of inflammation. Although CEH is not a neoplastic disease, six cases of angiosarcoma originating from CEHs have been reported [8]. Therefore, explorative surgery should be performed for definitive diagnosis. In our case, the findings were characteristic of CEH, and it was suspected even before surgery. However, it was difficult to exclude malignant diseases because of the rarity of CEHs in the liver. The main characteristic of CEH is exclusion symptoms caused by an enlarging mass. Therefore, CEH on the limbs and body surface is likely to be detected relatively early, while that in the thoracic or abdominal cavities may only be detected after forming a giant mass. There are some reports of ruptured cysts leading to a critical patient condition [9–12]. In our case, no serious exclusion symptoms were observed, but the imaging examination revealed findings, such as exclusion of the IVC and elevation of the right diaphragm due to the tumor. In addition, respiratory function examination showed a restraining pattern. Treatment of CEH involves complete removal of the tumor, including the cyst wall, to prevent recurrence, because it is highly possible that the fluid in the cyst will re-accumulate if only puncturing and drainage are performed [3, 13, 14]. In our case, the inside of the tumor was divided into several compartments by a blood clot, meaning that symptomatic improvement would have been unlikely by simple puncture and drainage. Nishida et al. [6] stated that some cases have strong adhesion to organs and tissues in contact with the cyst because of strong inflammation around the cyst margin. In such cases, combined resection of the organ [7, 15–17] or incomplete resection of the remnant cyst wall [18, 19] is unavoidable. In our case, we could dissect the extensive adhesions between the inferior margin of the tumor and the hepatic flexure. Even though the IVC was strongly excluded from the dorsal side on imaging, there were few intraoperative findings suggestive of inflammatory changes, such as fibrosis and IVC scarring. Since the CEH occurred in the liver, we believe that strong adhesions would have been unlikely to form given that the liver parenchyma was covered by the cyst wall (Fig. 4b). However, it is necessary to accumulate more cases to conduct a detailed examination of the degree of adhesion between the surrounding organs and CEH in the liver. Incomplete resection of CEH can cause major bleeding from the cyst wall, which is rich in blood flow [20]. In cases with strong adhesions to the surrounding tissues, massive bleeding may occur due to cyst wall damage. In cases with a large tumor diameter and a confirmed feeder artery in the cyst, preoperatively embolizing the feeder artery can reportedly block the inflow of blood and reduce tumor size, thus minimizing intraoperative bleeding [3, 21, 22]. By estimating the risk of bleeding using preoperative imaging examination of the tumor size, degree of inflammation, and blood flow findings on the cyst wall, we can consider additional preoperative treatment for cases with a high risk of bleeding.

## Conclusions

We encountered a rare case of CEH in the liver. This is only the second report of CEH in the liver worldwide. Accumulation of more cases in the future will further elucidate the pathological condition of CEH.

## Data Availability

Not applicable.

## Abbreviations

CEH: Chronic expanding hematoma
CT: Computed tomography
MRI: Magnetic resonance imaging
IVC: Inferior vena cava
FDG: Fluorodeoxyglucose
PET: Positron emission tomography
